# The Aberrant Behavior Checklist in a Clinical Sample of Autistic Individuals with Intellectual Disabilities and Co-Occurring Mental Health Problems: Psychometric Properties, Factor Structure, and Longitudinal Measurement Invariance

**DOI:** 10.1007/s10803-024-06697-5

**Published:** 2025-01-04

**Authors:** Arvid Nikolai Kildahl, Jane Margrete Askeland Hellerud, Marianne Berg Halvorsen, Sissel Berge Helverschou

**Affiliations:** 1 Regional Section Mental Health, Intellectual Disabilities/Autism, Oslo University Hospital, Dikemarksveien 39, Asker, 1385 Norway; 2 NevSom – Norwegian Centre of Expertise for Neurodevelopmental Disorders and Hypersomnias, Oslo University Hospital, Oslo, Norway; 3 Department of Pediatric Rehabilitation, University Hospital of North Norway, Tromsø, Norway

**Keywords:** Aberrant Behavior Checklist, Autism, Intellectual disability, Challenging behaviour, Assessment, Measurement invariance

## Abstract

The Aberrant Behavior Checklist (ABC) was originally developed to evaluate interventions, and is a well-established assessment tool for challenging behaviours in people with intellectual disabilities and autistic people. However, whether the ABC displays longitudinal measurement invariance (i.e., whether it captures the same constructs over time) has been sparsely explored. The aim of the current study is to explore the factor structure, longitudinal measurement invariance, and clinical correlates of the ABC in autistic individuals with intellectual disabilities. Using data from a multicentre study of mental health assessment and treatment in autistic people with intellectual disabilities, the intake ABC scores of 200 autistic individuals with intellectual disabilities were used to explore the ABC factor structure, internal consistency, and clinical correlates (age, gender, level of intellectual disability, autism characteristics, communication skills). Scores across three time points (intake, post-intervention, follow-up) were used to explore longitudinal measurement invariance and internal consistency over time. The original five-factor structure showed a non-optimal but acceptable fit, which was similar or slightly improved compared to previous studies. Associations for some ABC subscales were found to be positive for autism characteristics and negative for communication skills. Four of the five subscales (irritability, social withdrawal, stereotypic behaviour, inappropriate speech) showed residual levels of longitudinal measurement invariance, while one subscale showed noninvariance (hyperactivity/noncompliance). The current study demonstrates the construct validity and applicability of the ABC in autistic individuals with intellectual disabilities, while also indicating that caution is advised for one of its subscales in comparisons across time.

## Background

Emerson (1995) defines “challenging behaviours” as

…culturally abnormal behaviour(s) of such an intensity, frequency or duration that the physical safety of the person or others is likely to be placed in serious jeopardy, or behaviour which is likely to seriously limit use of, or result in the person being denied access to, ordinary community facilities (p. 3).

In line with Emerson’s definition, “challenging behaviours” is used as a descriptive term (Emerson, 1995) in the current paper and denotes behaviours that involve a risk to the safety of the person or others, or which result in limiting the person’s access to community facilities. The term likely encompasses a heterogeneous range of phenomena, including self-injurious and aggressive/disruptive behaviours, behaviours that the individual is unable to manage themselves (e.g. meltdowns), or autism-related restrictive/repetitive behaviours that involve risk of harm or result in negative social consequences (Bottema-Beutel et al., 2021; Bowring et al., 2019).

Autistic people with intellectual disabilities appear to be at high risk of developing challenging behaviours (Bowring et al., 2019; Horovitz et al., 2013; McCarthy et al., 2010; Sappok et al., 2014). The assessment of co-occurring difficulties in this population is complex (Bakken et al., 2016; Deb et al., 2022; Hollocks et al., 2019; Kildahl et al., 2024; Underwood et al., 2010, 2015), and often relies on informant reports and the integration of multiple sources of information (Deb et al., 2022; Kildahl et al., 2024). In people with intellectual disabilities more generally, previous research has found challenging behaviours to be associated with trait factors, such as emotional development and dysregulation (Melville et al., 2016; Sappok et al., 2014), sensory impairment (de Winter et al., 2011), and communication difficulties (Bowring et al., 2017; McClintock et al., 2003). Moreover, associations have been identified with state factors involving likely discomfort or distress, including mental health symptoms (Appleton et al., 2019; Bowring et al., 2019; Kildahl et al., 2023; Painter et al., 2018; Peña-Salazar et al., 2022), pain and somatic disease (Bowring et al., 2019; Leader et al., 2021; de Winter et al., 2011), as well as potentially traumatic experiences (Brenner et al., 2018; Kildahl & Helverschou, 2024; Rittmannsberger et al., 2020). Finally, several studies have identified associations with environmental factors (Bowring et al., 2019; Emerson, 1995), including service provider behaviour (Wolkorte et al., 2019). Thus, challenging behaviours appear to constitute a multifaceted and complex phenomenon with numerous risk factors across different domains, in which risk factors may be related, affect each other, or even show a bidirectional relationship with challenging behaviours (Bowring et al., 2019; Felce & Kerr, 2013; Kildahl & Helverschou, 2024; Kildahl et al., 2023).

Originally developed as a measure to monitor the effects of treatment in people with intellectual disabilities (Aman, 2012; Aman et al., 1985; Aman & Singh, 1986), the Aberrant Behavior Checklist (ABC) remains one of the most well-studied informant-completed checklists for the assessment of challenging behaviours in people with intellectual disabilities and autistic people (Aman, 2012; Halvorsen et al., 2022, 2023, 2024; Helverschou et al., 2020). The ABC comprises 58 items, describing a range of behaviours that may be perceived as challenging by others. Item ratings are used to calculate scores for five empirically constructed subscales: irritability, social withdrawal, stereotypic behaviour, hyperactivity/noncompliance, and inappropriate speech.

Studies of the ABC’s psychometric properties have shown good to excellent reliability across varying levels of intellectual disabilities (Aman et al., 2012; Flynn et al., 2017; Halvorsen et al., 2023), as well as in autistic children and adolescents (Brinkley et al., 2007; Halvorsen et al., 2024; Kaat et al., 2014; Norris et al., 2019). A recent systematic review of the general tools used in mental health assessment of autistic people concluded that the ABC was the only assessment tool to show good/excellent properties for all aspects of reliability, including internal consistency, test-retest reliability, and interrater reliability (Halvorsen et al., 2024). However, there have been few studies of the psychometric properties of the ABC in autistic adults with co-occurring intellectual disabilities, as most studies thus far have been on children and adolescents (Halvorsen et al., 2024), or non-autistic adults with intellectual disabilities (Flynn et al., 2017). Moreover, some studies have found a slightly different factor structure in autistic samples, with the three items describing self-injurious behaviour emerging as a separate factor and not as part of the irritability subscale, as in the original factor structure (Brinkley et al., 2007; Kaat et al., 2014; Halvorsen et al., 2019).

The ABC – occasionally the irritability subscale only (Stoddard et al., 2020) – is frequently used in research evaluating interventions and treatment, including pharmacological treatment, for people with intellectual disabilities (Aman, 2012; Aman et al., 2010; Stoddard et al., 2020). Longitudinal measurement invariance concerns whether scores from an assessment tool are comparable in the same sample over time (Mackinnon et al., 2022; Putnick & Bornstein, 2016). In spite of its extensive use in treatment evaluation, we are not aware of any previous exploration of the measurement invariance of the ABC. An exploration of longitudinal measurement invariance is essential to determine whether scores across time reflect the same underlying phenomenon, and thus whether they are comparable and can be used as a basis for conclusions regarding the measured effects of interventions.

Finally, there is a lack of research exploring the factor structure and psychometric properties of the non-English versions of the ABC (Aman, 2012). The findings regarding the Norwegian version of the ABC have been positive when it comes to reliability and convergent validity (Bakken et al., 2024; Halvorsen et al., 2019; Helverschou et al., 2021; Kildahl et al., 2023; Myrbakk & von Tetzchner, 2008a; Myrbakk & Tetzchner, 2008b), although thus far its factor structure has only been investigated in a neuropediatric sample (Halvorsen et al., 2019).

In the current study, we aim to explore the psychometric properties of the Norwegian version of the ABC, including factor structure and measurement invariance in autistic people across time. For this purpose, we will use data from a clinical sample of autistic individuals with intellectual disabilities, predominantly adults, who were referred to specialist health services for the assessment of mental health and/or challenging behaviours. The research questions were as follows:

1. Does the original ABC factor structure show acceptable fit in autistic individuals with intellectual disabilities at intake?
2. What is the internal consistency of the ABC subscales at intake?
3. What is the relationship between ABC subscale scores at intake and age, gender, level of intellectual disability, autism characteristics, and communication skills?
4. Do the ABC subscales demonstrate longitudinal measurement invariance during the treatment of mental health disorder in autistic individuals with intellectual disabilities?
5. What is the internal consistency of the ABC subscales with repeated use in the same sample over time?

## Methods

### Design and Setting

The current study uses data from the Norwegian AUP (Autism, Intellectual Disability, Mental Illness) study, a nation-wide longitudinal multi-centre study of the assessment/treatment of mental health disorders in autistic individuals with intellectual disabilities (Helverschou et al., 2021). Eight centres providing specialist mental health services for this population throughout Norway participated in the study, and collected data from 2010 to 2020. Data were collected for each participant at three time points: at intake/pre-intervention (T1), after 12 months/post-intervention (T2), and after 24–27 months/follow-up (T3). The eight centres are organised in different ways, and chose potential participants for inclusion (Helverschou et al., 2021). All the centres have outpatient services, while two also have inpatient facilities. For a further description of the study and the centres, see Helverschou et al. (2021).

### Participants

Prior to inclusion, all participants had been diagnosed with autism spectrum disorder (ASD) and intellectual disability according to the ICD-10 (World Health Organization, 1992). In addition, the inclusion criteria included suspected mental health disorder or challenging behaviour, as well as being referred to one of the eight centres. No exclusion criteria were applied with regard to further co-occurring disorders or disabilities. Diagnoses of ASD and intellectual disability were independently reviewed and verified by a psychiatrist/clinical psychologist. While autism diagnostic tools were not part of the AUP study protocol, the study included the Social Communication Questionnaire (SCQ; Rutter et al., 2003), current and lifetime versions, for the measurement of autism characteristics. For those participants who scored below the recommended cut-off (> 15) on the SCQ current version, the lifetime version was consulted. For those participants who also scored below the recommended cut-off on the lifetime version, or where this was missing, the respective centres were contacted to ensure that the participant in question had been diagnosed with autism. This was confirmed for all the included participants.

The participating centres differ somewhat in their organisation and target groups. While some centres provide services for adults only (age > 18), some also include adolescents (age > 14 or age > 16), while one centre provides services to children. For the current study, all participants in the data set over the age of 13 were included.

For the cross-sectional aspects of the study (research questions 1–3), all participants with completed ABCs at T1 were included. The sample size was 200 participants (59 female, 141 male), all diagnosed with autism, aged 13–68 (*M* = 28.67, *SD* = 11.43). Of these, 128 were diagnosed with mild/moderate intellectual disabilities, 71 with severe/profound intellectual disabilities, while these data were missing for 1 participant.

Because the AUP is a clinical study organised as a part of regular service delivery, there was some attrition in the sample. For the longitudinal aspects (research questions 4–5), the sample included all participants with completed ABCs from all three time points: 169 participants (55 female, 114 male; 107 mild/moderate, 62 severe/profound), all diagnosed with autism, aged 13–68 (*M* = 28.27, *SD* = 11.16).

### Measures

#### Aberrant Behavior Checklist – Community Version (ABC)

The ABC is a proxy-rated checklist for challenging behaviours in people with intellectual disabilities (Aman, 2012; Aman et al., 1985; Aman & Singh, 1986, 2017). The ABC comprises 58 items scored on a 4-point Likert scale (0 = no problem, 1 = minor problem, 2 = moderate problem, 3 = severe problem). Item scores are summed into five subscales: irritability (15 items), social withdrawal (16 items), stereotypic behaviour (7 items), hyperactivity/noncompliance (16 items), and inappropriate speech (4 items). The ABC is a dimensional measure, and higher scores on the respective subscales reflect more severe levels of challenging behaviour within these domains.

The ABC has been widely used and studied (Aman, 2012; Halvorsen et al., 2022, 2023, 2024; Helverschou et al., 2020) and appears to be one of the most thoroughly researched checklists for autistic people (Halvorsen et al., 2024) and people with intellectual disabilities (Flynn et al., 2017; Halvorsen et al., 2023; Helverschou et al., 2020). Its psychometric properties have been found to be good to excellent in these populations (Aman, 2012; Flynn et al., 2017; Halvorsen et al., 2022, 2023, 2024). Similar findings have been made for the Norwegian version in a neuropediatric sample (Halvorsen et al., 2019).

#### Social Communication Questionnaire (SCQ)

The social communication questionnaire (SCQ; Rutter et al., 2003), current version, was used for the measurement of autism characteristics. The SCQ is a proxy-completed checklist comprising 40 yes/no items describing autism characteristics, and has been found to have good psychometric properties in identifying autism (Berument et al., 1999), including in adults with intellectual disabilities (Wigham et al., 2019). There are no separate norms for the Norwegian version and it is used and interpreted in the same way as the original version (Kaasbøll et al., 2021).

#### Vineland Adaptive Behavior Scales-Communication (VABS-C)

The participants’ levels of communication skills were measured using the standard scores from the communication scale of the Vineland Adaptive Behavior Scales, second edition, expanded version (Sparrow et al., 2008). The VABS have been extensively used in research and clinical practice for decades, and remain the adaptive behaviour assessment tool that is supported by the most extensive body of research (Floyd et al., 2015).

#### Level of Intellectual Disability

In the AUP study, level of intellectual disability was reported by clinicians as a dichotomous variable, as mild/moderate or severe/profound. Due to difficulties in obtaining reliable IQ scores from participants with severe behavioural and/or mental health problems, intellectual disability diagnoses were confirmed by clinical judgement, which was informed by clinical background information from hospital records, previous diagnoses, VABS scores (Sparrow et al., 2008), and behavioural observation by expert clinicians.

### Procedure

Data for the AUP study were collected as part of regular service delivery, with T1 data collected at intake, T2 at the end of treatment or after 12 months, and T3 data at follow-up after another year (see Helverschou et al., 2021). The eight participating centres chose which patients to include in the study, according to their capacity and organisation. The ABC, SCQ, and VABS were completed by a professional caregiver from the participant’s residential care home (ABC T1: 88.5%), or a family member (ABC T1: 10%; missing: 1.5%).

### Research Ethics

The AUP multi-centre study was approved by the Data Protection Officer, Oslo University Hospital, Oslo, Norway, #2010/19579. Due to the participants’ limited ability to consent, informed consent was obtained from the legal guardians of all the participants. The participants also consented themselves, when this was feasible. All data were de-identified prior to submission from each centre.

### Analyses

The Statistical Package for Social Sciences 29 was used for all analyses, except for the confirmatory factor analyses (CFA) and the analyses of measurement invariance (MI), which were conducted in R, using lavaan (Rosseel, 2012) and semTools (Jorgensen et al., 2022). Missing items were managed subscale-wise, by imputation of the mean item score for the remaining items on the respective subscale, in line with the recommendations of the ABC-2 manual (Aman & Singh, 2017). To allow for the use of analytic strategies for ordinal data, imputed values were rounded up or down to the closest whole integer. If more than the prescribed number of items for any subscale were missing (3 for irritability, social withdrawal, hyperactivity/noncompliance; 2 for stereotypic behaviour; 1 for inappropriate speech), the participant was excluded. The number of substitutions for the cross-sectional analysis using T1 data were 30 (0.26% of item scores), while the substitutions for the longitudinal analysis were 24 for T1 (0.24%), 17 for T2 (0.17%), and 25 for T3 (0.26%).

To explore the potential consequences of attrition, comparisons between the longitudinal sample (*N* = 169) and the participants included only in the cross-sectional aspect of the study due to missing data from either T2, T3, or both (*N* = 31), were conducted for all variables. Welch’s ANOVA (age, ABC subscale scores, SCQ, VABS-C) and Fisher’s exact test (gender, level of intellectual disability) were used for these analyses.

#### Factor Analysis, Reliability, and Covariates

CFAs were conducted in R, using lavaan (Rosseel, 2012). Because item scores in the ABC are scored using a 4-point Likert scale, diagonally weighted least squares (DWLS; Li, 2016) were used to estimate the model parameters and fit indices. CFAs were conducted for a one-factor model, the original five-factor model, and a six-factor model based on previous findings from autistic samples identifying the three items describing self-injurious behaviour as a separate factor. Due to non-optimal fit, a post hoc exploratory factor analysis (EFA) was conducted in R, based on the polychoric correlation matrix due to the data being ordinal (Holgado-Tello et al., 2010), and using Varimax rotation.

McDonald’s omega (*ω*) was used to calculate internal consistency (Hayes & Coutts, 2020; McDonald, 1999). Omega was preferred to Cronbach’s alpha, because the factor loadings are used in its calculation, allowing the associations between the latent variable and the items to vary. Associations with age, autism characteristics (SCQ), and communication skills (VABS-C) were investigated using Spearman’s rho, while differences in scores according to gender or level of intellectual disability were explored using Welch’s ANOVA (Delacre et al., 2019). To account for the unique variance associated with each covariate, multiple linear regression models were calculated with each ABC subscale as the dependent variable, and age, gender, level of intellectual disability, autism characteristics (SCQ), and communication skills (VABS-C) as predictors. To investigate potential two-way interactions between the covariates, standardised interaction terms for covariates found to be associated with the respective ABC subscales in the univariate analyses were explored. Interaction terms showing a significant effect on the dependent variable when entered separately were retained in the final models.

#### Measurement Invariance

Measurement invariance was explored using a number of increasingly constrained multi-group CFAs (Putnick & Bornstein, 2016), referred to as nested models. These explorations are typically conducted in four steps (Putnick & Bornstein, 2016): configural, metric, scalar, and residual. Configural invariance involves establishing the equivalence of model form, i.e. whether the measured factors have the same pattern of free and fixed factor loadings. Recent recommendations for testing configural invariance involve the use of permutation tests (Kite et al., 2018; Jorgensen et al., 2017, 2018). Metric invariance involves establishing equivalence of item loadings by constraining factor loadings across the two groups and comparing model fit to the configural model. Scalar invariance involves establishing equivalence of item intercepts, i.e. “that mean differences in the latent construct capture all mean differences in the shared variance of items” (Putnick & Bornstein, 2016, p. 76), by constraining item intercepts to be equal across the two groups and comparing model fit with the metric model. Scalar invariance is the level of invariance required for comparison of means between the two groups. Residual invariance involves establishing that the sum of specific and error variance is similar across the groups. Significant changes in fit indices with increasing constraints indicate a lack of invariance (Putnick & Bornstein, 2016). If invariance cannot be established during any of these steps, the analysis is either discontinued or the model is adjusted to establish partial invariance, for example, by releasing constraints on certain parameters of the model (Putnick & Bornstein, 2016).

For categorical and ordinal data, including Likert-scale type responses such as the ABC, Wu and Estabrook (2016) have shown that it is not possible to constrain factor loadings in the investigation of metric invariance without also constraining the thresholds between the two groups. We therefore followed the recommendations of Wu and Estabrook (2016; see also Padgett, 2023) and created a separate nested model in which the thresholds were constrained prior to constraining the item loadings. Thus, in the current study, investigation of measurement invariance comprised five steps: configural invariance, threshold invariance, metric invariance, scalar invariance, and residual invariance. Theta parameterization and DWLS were used for these analyses, and calculation of the *χ*^*2*^ difference between the nested models was conducted using the procedure recommended by Satorra (2000).

Different strategies have been reported for the evaluation of relative fit of nested models (Putnick & Bornstein, 2016), Padgett, (2023) has posited that researchers should consider “the breadth of information across indices and tests to make an informed decision about the tenability of invariance” (p. 6). *χ*^*2*^ difference has been commonly used, but may be sensitive to measurement parameters that are not necessarily relevant to the latent (factor) estimate (Jorgensen et al., 2018; Putnick & Bornstein, 2016). Cheung and Rensvold (2002) suggested using ΔCFI of − 0.01 as a cutoff value for nested models. Specifically for smaller samples with unequal sample sizes, Chen (2007) suggested a more conservative − 0.005 cutoff for ΔCFI, in combination with either changes to RMSA of ≥ 0.010 or changes to SRMR of ≥ 0.025 (metric invariance) or ≥ 0.005 (scalar/residual invariance).

Because the ABC has an empirically established factor structure that was used in these analyses, we chose to accept non-optimal fit for the non-restricted CFAs, rather than altering the original ABC subscales to improve statistical fit and establish partial invariance. The ABC is frequently used in research and clinical settings for the evaluation of treatment/intervention effects. Thus, knowledge concerning the measurement invariance of the original factor structure is essential to evaluate the appropriateness of this use. For the configural models, we chose to use permutation tests (3000 permutations) for evaluating fit (Jorgensen et al., 2018; Kite et al., 2018), in line with the strategy described by Padgett (2023) using nonparametric permutation test *p*-values for *χ*^*2*^, CFI, RMSEA, and SRMR obtained via the permuteMeasEq function of semTools in R (Jorgensen et al., 2022). For the remaining models (threshold, metric, scalar, residual), we chose to use the cutoffs suggested by Chen (2007). While this may involve a risk of Type I error (Jorgensen et al., 2018; Kite et al., 2018), the current study involved a high number of models and analytic steps, and the Chen (2007) criteria allowed for a more parsimonious analytic strategy.

Because some ABC subscales have been used as standalone measures (Stoddard et al., 2020), and are to be interpreted individually (Aman, 2012; Aman & Singh, 2017), we specified nested path models for each ABC subscale, in line with the strategy used by Dovgan et al. (2019) for the Child Behavior Checklist, which is interpreted in a similar way. For the permutation test of equivalence of model form (configural invariance), the three time points were treated as separate groups. The longitudinal function of the measEq.syntax was used for the remaining models, including for calculating the fit indices of the configural invariance model. Because configural noninvariance was identified for one of the subscales, post hoc EFAs were conducted for T2 and T3 data, to explore potential variations in model form across time. As for T1 data, this analysis was based on the polychoric correlation matrix and using Varimax rotation.

## Results

### Does the Original ABC Factor Structure Show Acceptable Fit in Autistic Individuals with Intellectual Disabilities at Intake?

CFA was conducted for a one-factor model, *χ2*(1595, *N* = 200) = 5246.31, *p* <.001, (RMSEA = 0.107, RMSEA 90% CI [0.104, 0.110], TLI = 0.686, CFI = 0.697, GFI = 0.827, AGFI = 0.802), and the original five-factor model, *χ2*(1585, *N* = 200) = 2946.36, *p* <.001, (RMSEA = 0.066, RMSEA 90% CI [0.062, 0.069] TLI = 0.882, CFI = 0.886, GFI = 0.929, AGFI = 0.918). The fit indices for the one-factor solution were poor. While the fit indices for the five-factor solution were better, and a considerable improvement on the one-factor solution, they remained slightly below the recommended values. CFA was also conducted for a six-factor solution with the three items describing self-injurious behaviour as a separate factor. Fit indices were slightly improved from the five-factor solution, *χ2*(1580, *N* = 200) = 2708.88, *p* <.001, (RMSEA = 0.060, RMSEA 90% CI [0.056, 0.065], TLI = 0.902, CFI = 0.906, GFI = 0.941, AGFI = 0.931).

Due to the non-optimal fit, a post-hoc EFA was conducted, with the Scree Plot parallel analysis identifying seven underlying factors, explaining 59.8% of variance. These included the original stereotypic behaviour (F4 in this model, F3 in the original) and inappropriate speech (F7 in this model, F5 in the original) factors, as well as a separate factor for self-injurious behaviour (F6). The items describing non-compliant or disruptive behaviours from the original hyperactivity/non-compliance subscale loaded with the items from the original irritability factor (F1 in this model and in the original) and excessive motor activity/restlessness emerged as a separate factor (F2 in this model). The original factor 2 (social withdrawal) emerged as two separate factors, one describing social withdrawal (F3) and one describing lethargic/passive behaviour (F6). Notably, 17/58 items loaded > 0.30 on more than one factor. These included cross-loadings between the irritability and self-injurious behaviour factors, as well as between the irritability and excessive motor activity/restlessness factors. In addition, cross-loadings were found for the two factors encompassing items from the original social withdrawal subscale. One item, “lacks emotional response” loaded on three factors: social withdrawal, lethargic/passive behaviour, and stereotypic behaviour. CFA based on the alternative model identified in the EFA resulted in a slightly better fit than the six-factor solution, *χ2*(1463, *N* = 200) = 2227.56, *p* <.001, (RMSEA = 0.051, RMSEA 90% CI [0.047, 0.056], TLI = 0.932, CFI = 0.935, GFI = 0.958, AGFI = 0.951).

**Table 1:** Ranges, mean scores, and standard deviations for ABC subscales

Time	Irritability		Social withdrawal		Stereotypic behaviour		Hyperactivity/ noncompliance		Inappropriate speech	
	Range	*M* (*SD*)	*Range*	*M* (SD)	*Range*	*M* (*SD*)	*Range*	*M* (*SD*)	Range	*M* (*SD*)
*Full sample at T1 (n* *= 200)*										
T1	0–45	16.94 (11.12)	0–38	14.52 (9.55)	0–21	5.74 (5.04)	0–46	16.64 (10.96)	0–12	4.39 (3.65)
*Longitudinal sample across T1-T3 (n* *= 169)*										
T1	0–45	17.10 (11.20)	0–38	14.72 (9.60)	0–21	5.69 (5.04)	0–46	17.03 (11.24)	0–12	4.27 (3.68)
T2	0–42	12.59 (10.04)	0–37	10.69 (7.71)	0–21	4.11 (4.37)	0–43	11.27 (9.04)	0–12	2.99 (3.04)
T3	0–35	11.71 (9.39)	0–39	9.90 (8.06)	0–18	3.66 (4.02)	0–37	10.45 (8.88)	0–12	3.12 (2.93)

Note Aberrant Behavior Checklist (ABC). All subscales have a minimum score of 0. Maximum scores are 48 for social withdrawal and hyperactivity/non-compliance, 45 for irritability, 21 for stereotypic behaviour, and 12 for inappropriate speech

### What is the Internal Consistency of the ABC Subscales at Intake?

Ranges, mean scores, and standard deviations for the original ABC subscales in the sample at T1 are presented in Table 1. The internal consistency of the ABC subscales at intake is reported in Table 2.

**Table 2:** Internal consistency (McDonald’s ω) for ABC subcales

Time	Irritability	Social withdrawal	Stereotypic behaviour	Hyperactivity/ noncompliance	Inappropriate speech
*Full sample at T1 (n* *= 200)*					
T1	0.91	0.88	0.86	0.90	0.81
*Longitudinal sample across T1-T3 (n* *= 169)*					
T1	0.91	0.87	0.86	0.90	0.82
T2	0.92	0.85	0.86	0.91	0.80
T3	0.90	0.88	0.84	0.90	0.77

Note Aberrant Behavior Checklist (ABC)

### What is the Relationship Between ABC Subscale Scores at Intake and Age, Gender, Level of Intellectual Disability, Autism Characteristics, and Communication Skills?

In the univariate analyses, the irritability subscale showed significant negative associations with age *r*(200) = -14, *p* =.046 and the VABS-C, *r*(198) = − 0.26, *p* <.001, as well as a significant positive association with the SCQ, *r*(198) = 0.26, *p* <.001. Also, participants with severe/profound intellectual disabilities (*M* = 20.20, *SD* = 10.75) had higher scores than those with mild/moderate intellectual disabilities (*M* = 15.24, *SD* = 10.94), *F* = 9.58(1, 146.78), *p* =.002. No significant difference was found for gender. In the multiple regression model, only the SCQ score remained a significant predictor of the irritability subscale score, and no significant interactions were identified, see Table 3.

**Table 3:** Multiple regression for age, gender, level of intellectual disability, SCQ, VABS-C on ABC subscales (*N* = 200)

Predictors	*Unstandardized coefficients*			*Standardized coefficients*					
	*B*	*SE*	*95% CI*	*ß*	*p*	*R* ^*2*^	*Adjusted R* ^*2*^	*F*	*p*
*Irritability*						0.126	0.103	5.47	< 0.001
Age	-0.12	0.07	[-0.25, 0.01]	-0.12	0.080				
Gender	-2.64	1.66	[-5.93, 0.64]	-0.11	0.114				
Level of intellectual disability	2.22	2.07	[-1.87, 6.31]	0.10	0.286				
SCQ	0.31	0.14	[0.03, 0.58]	0.17	0.028*				
VABS-C	-0.09	0.07	[-0.23, 0.05]	-0.13	0.204				
*Social withdrawal*						0.138	0.116	6.07	< 0.001
Age	-0.05	0.06	[-0.16, 0.06]	-0.06	0.349				
Gender	0.26	1.40	[-2.50, 3.03]	0.01	0.851				
Level of intellectual disability	0.54	1.75	[-2.91, 3.98]	0.03	0.759				
SCQ	0.59	0.18	[0.36, 0.83]	0.39	< 0.001***				
VABS-C	0.05	0.06	[-0.07, 0.17]	0.08	0.421				
*Stereotypic behaviour*						0.236	0.216	11.68	< 0.001
Age	-0.03	0.03	[-0.09. 0.02]	-0.07	0.271				
Gender	0.52	0.70	[-0.86, 1.91]	0.05	0.458				
Level of intellectual disability	0.86	0.87	[-0.87, 2.58]	0.08	0.328				
SCQ	0.25	0.06	[0.14, 0.37]	0.31	< 0.001***				
VABS-C	-0.06	0.03	[-0.12, -0.00]	-0.20	0.035*				
*Hyperactivity/noncompliance*						0.157	0.126	4.99	< 0.001
Age	-0.20	0.45	[-1.09, 0.69]	-0.21	0.657				
Gender	2.44	1.63	[-1.09, 5.66]	0.10	0.137				
Level of intellectual disability	-2.24	5.53	[-13.14, 8.67]	-0.10	0.686				
SCQ	0.16	0.14	[-0.11, 0.43]	0.09	0.236				
VABS-C	0.01	0.17	[-0.33, 0.35]	0.02	0.951				
VABS-C x age	-3.14	3.67	[-10.38, 4.09]	-0.29	0.392				
Level of intellectual disability x age	3.92	4.09	[-4.14, 11.98]	0.36	0.339				
*Inappropriate speech*						0.041	0.016	1.63	0.155
Age	0.00	0.02	[-0.04, 0.05]	0.00	0.968				
Gender	0.27	0.57	[-0.85, 1.40]	0.03	0.631				
Level of intellectual disability	-0.04	0.71	[-1.44, 1.36]	-0.01	0.959				
SCQ	0.13	0.05	[0.03, 0.22]	0.22	0.008**				
VABS-C	0.01	0.02	[-0.04, 0.06]	0.04	0.738				

Note Aberrant Behavior Checklist (ABC), Social Communication Questionnaire (SCQ), Vineland Adaptive Behavior Scales-Communication (VABS-C). *p = < 0.05, **p = < 0.01, ***p = < 0.001

The social withdrawal subscale showed a significant positive association with the SCQ score, *r*(198) = 0.30, *p* <.001, in the univariate analyses. No associations were found for age or the VABS-C, nor any significant differences according to gender or level of intellectual disability. In the multiple regression model, the SCQ score remained a significant predictor of score on the social withdrawal subscale.

For stereotypic behaviour, univariate analyses identified a significant positive association with SCQ, *r*(198) = 0.42, *p* <.001, and a significant negative association with VABS-C, *r*(198) = − 0.37, *p* <.001. Moreover, participants with severe/profound intellectual disabilities had higher scores on this subscale (*M* = 7.59, *SD* = 5.50) than those with mild/moderate intellectual disabilities (*M* = 4.76, *SD* = 4.47), *F* = 13.79(1, 121.57), *p* <.001. No association was found for age, nor any significant differences according to gender. In the multiple regression model, both the SCQ and VABS-C scores remained significant as predictors of the stereotypic behaviour score, and no significant interactions were identified.

The univariate analyses for hyperactivity/noncompliance identified a significant positive association with the SCQ, *r*(198) = 0.20, *p* =.004, and significant negative associations with age, *r*(200) = − 0.18, *p* =.011, and the VABS-C, *r*(198) = − 0.29, *p* <.001. In addition, participants with severe/profound intellectual disabilities had higher scores (*M* = 20.56, *SD* = 11.59) on hyperactivity/noncompliance than participants with mild/moderate intellectual disabilities (*M* = 14.56, *SD* = 10.01), *F* = 13.47(1, 127.80), *p* <.001. No significant differences were identified according to gender. Two potential interactions were identified (VABS-C x age, level of intellectual disability x age). However, these were not significant when both interactions were included in the model. While the entire final model remained significant, no predictor was significant, see Table 3.

For inappropriate speech, no significant differences were found for age or level of intellectual disability. A positive association was only identified for the SCQ, *r*(198) = 0.20, *p* =.006, and no association was identified for age or the VABS-C. The multiple regression model was not significant, even if the SCQ emerged as a significant predictor.

### Do the ABC Subscales Demonstrate Longitudinal Measurement Invariance During Treatment of Mental Health Disorder in Autistic Individuals with Intellectual Disabilities?

Due to attrition, the longitudinal sample differed significantly from the cross-sectional sample with regard to participant gender (*p* =.032), with the attrition being proportionally higher among male participants (27/141) than among female participants (4/59). There was no significant difference between the samples for the cross-sectional and longitudinal aspects of the study with regard to level of intellectual disability or age, nor for scores on the SCQ, VABS-C, and the ABC subscales.

In the analysis of longitudinal measurement invariance, fit indices for the configural models for all subscales were similar to the fit indices of the CFA, and the permutation tests indicated equivalence of model form across the three time points for four of the subscales (irritability, social withdrawal, stereotypic behaviour, inappropriate speech), see Table 4. The permutation test for hyperactivity/noncompliance indicated a lack of equivalence of model form, and investigation of measurement invariance was discontinued for the remaining models. For the remaining four subscales, analyses indicated residual levels of measurement invariance across the three time points in the current sample.

**Table 4:** Nested Model Comparisons of ABC Subscales Across T1-T3 (*N* = 169)

Model	χ2 (df)	CFI	TLI	RMSEA [90% CI]	SRMR	GFI	AGFI	Δχ^2^ (Δ*df*)	ΔCFI	ΔRMSEA	ΔSRMR	Decision
*Irritability*												
CFA T1	474.86 (90)***	0.944	0.935	0.160 [0.146, 0.174]	0.142	0.975	0.958					
CFA T2	392.09 (90)***	0.941	0.931	0.141 [0.127, 0.156]	0.119	0.977	0.962					
CFA T3	474.00 (90)***	0.933	0.922	0.159 [0.145, 0.174]	0.160	0.966	0.943					
Configural invariance	1689.48 (897)***	0.940	0.934	0.073 [0.067, 0.078]	0.112	0.966	0.958					Accept
*Permutation test p-values*	0.998	0.262		0.998	0.836							
Threshold invariance	1726.61 (927)***	0.939	0.935	0.072 [0.066, 0.077]	0.112	0.966	0.959	33.96 (30)	− 0.001	-0.001	0.000	Accept
Metric invariance	1745.68 (955)***	0.940	0.938	0.070 [0.065, 0.075]	0.112	0.966	0.960	34.80 (28)	0.001	-0.001	0.000	Accept
Scalar invariance	1764.87 (983)***	0.941	0.940	0.069 [0.064, 0.074]	0.112	0.965	0.960	49.42 (28)**	0.001	-0.001	0.000	Accept
Residual invariance	1719.76 (1013)***	0.947	0.948	0.064 [0.059, 0.070]	0.114	0.963	0.959	36.72 (30)	0.006	-0.004	0.002	Accept
*Social withdrawal*												
CFA T1	431.64 (104)***	0.908	0.894	0.137 [0.124, 0.150]	0.130	0.959	0.933					
CFA T2	400.33 (104)***	0.915	0.901	0.130 [0.117, 0.144]	0.149	0.958	0.932					
CFA T3	293.08 (104)***	0.910	0.896	0.104 [0.090, 0.118]	0.117	0.968	0.949					
Configural invariance	1561.36 (1029)***	0.913	0.905	0.055 [0.050, 0.061]	0.121	0.937	0.922					Accept
*Permutation test p-values*	0.404	0.219		0.404	0.223							
Threshold invariance	1594.35 (1061)***	0.913	0.907	0.055 [0.049, 0.060]	0.121	0.937	0.924	25.10 (32)	0.000	-0.001	0.000	Accept
Metric invariance	1620.17 (1091)***	0.914	0.911	0.054 [0.048, 0.059]	0.121	0.935	0.924	43.61 (30)	0.001	-0.001	0.000	Accept
Scalar invariance	1633.96 (1121)***	0.916	0.916	0.052 [0.047, 0.058]	0.121	0.933	0.924	37.84 (30)	0.003	-0.002	0.001	Accept
Residual invariance	1666.17 (1153)***	0.916	0.918	0.051 [0.046, 0.057]	0.125	0.928	0.921	66.93 (32)***	0.000	-0.001	0.004	Accept
*Stereotypic behaviour*												
CFA T1	15.82 (14)	0.999	0.998	0.028 [0.000, 0.082]	0.041	0.998	0.993					
CFA T2	23.74 (14)*	0.993	0.990	0.064 [0.004, 0.108]	0.058	0.997	0.990					
CFA T3	26.58 (14)*	0.988	0.982	0.073 [0.027, 0.115]	0.064	0.994	0.983					
Configural invariance	180.56 (165)	0.995	0.994	0.024 [0.000, 0.043]	0.067	0.991	0.986					Accept
*Permutation test p-values*	0.327	0.265		0.327	0.380							
Threshold invariance	194.19 (179)	0.995	0.994	0.022 [0.000, 0.042]	0.067	0.991	0.986	12.01 (14)	0.000	-0.001	0.000	Accept
Metric invariance	204.65 (191)	0.996	0.995	0.021 [0.000, 0.040]	0.067	0.990	0.986	10.32 (12)	0.000	-0.002	0.000	Accept
Scalar invariance	218.41 (203)	0.995	0.995	0.021 [0.000, 0.040]	0.068	0.989	0.985	14.58 (12)	− 0.001	0.001	0.000	Accept
Residual invariance	233.40 (217)	0.995	0.995	0.021 [0.000, 0.040]	0.072	0.987	0.984	16.28 (14)	0.000	0.000	0.004	Accept
*Hyperactivity/noncompliance*												
CFA T1	551.24 (104)***	0.877	0.858	0.160 [0.147, 0.173]	0.152	0.946	0.913					
CFA T2	443.16 (104)***	0.886	0.868	0.139 [0.126, 0.153]	0.117	0.965	0.943					
CFA T3	430.36 (104)***	0.896	0.880	0.137 [0.123, 0.150]	0.129	0.960	0.936					
Configural invariance	1771.25 (1029)***	0.884	0.873	0.066 [0.060, 0.071]	0.113	0.943	0.930					Reject
*Permutation test p-values*	0.091	0.000		0.091	0.007							
*Inappropriate speech*												
CFA T1	4.71 (2)	0.997	0.990	0.090 [0.000, 0.198]	0.032	0.999	0.987					
CFA T2	4.41 (2)	0.996	0.988	0.085 [0.000, 0.194]	0.035	0.998	0.986					
CFA T3	9.35 (2)**	0.991	0.974	0.148 [0.062, 0.249]	0.055	0.998	0.979					
Configural invariance	67.74 (39)**	0.987	0.979	0.066 [0.039, 0.092]	0.061	0.993	0.982					Accept
*Permutation test p-values*	0.993	0.990		0.993	0.901							
Threshold invariance	78.54 (47)**	0.986	0.981	0.063 [0.037, 0.087]	0.061	0.993	0.984	10.31 (8)	− 0.001	-0.003	0.000	Accept
Metric invariance	91.60 (53)***	0.983	0.979	0.066 [0.042, 0.088]	0.063	0.991	0.983	14.42 (6)*	− 0.003	0.003	0.001	Accept
Scalar invariance	92.81 (59)**	0.985	0.983	0.058 [0.034, 0.080]	0.063	0.990	0.983	4.90 (6)	0.002	-0.007	0.001	Accept
Residual invariance	97.43 (67)**	0.987	0.987	0.052 [0.027, 0.073]	0.065	0.989	0.983	7.89 (8)	0.001	0.001	0.002	Accept

Note *p <.05, **p <.01, ***p <.001. Aberrant Behavior Checklist (ABC). Confirmatory Factor Analysis (CFA)

In light of the finding from the cross-sectional post-hoc EFA that hyperactivity/non-compliance subscale items did not load on a single factor, the lack of configural invariance for this subscale was explored by conducting post-hoc EFAs for the entire ABC at T2 and T3, see Appendices 2 and 3. At T2, a seven-factor solution was identified, while a five-factor solution was identified at T3. The factor structure at T2 appeared to be similar to the factor structure found in the post hoc analysis at T1. The original hyperactivity/non-compliance subscale items describing non-compliant or disruptive behaviours loaded with items from the original irritability factor (F1) and excessive motor activity/restlessness emerged as a separate factor (F2). However, at T3, most of the items from the original hyperactivity/non-compliance subscale appeared to load on a single factor.

### What is the Internal Consistency of the ABC with Repeated Use in the Same Sample Over Time?

Internal consistency remained similar across the three time points, except for inappropriate speech, for which it was slightly poorer at T3, see Table 2.

## Discussion

The current study found the ABC to have good to excellent reliability and demonstrated longitudinal measurement invariance for four of its five original subscales. These findings confirm that the ABC is an applicable assessment tool for autistic adults with intellectual disabilities. However, these findings highlight that caution is advised in the interpretation of the hyperactivity/noncompliance subscale when using the ABC to evaluate treatment and intervention.

The internal consistency of the ABC subscales was good to excellent across the three time points, in line with previous findings (Halvorsen et al., 2024). As for its factor structure, the current findings are in line with previous findings from autistic samples (e.g. Brinkley et al., 2007; Kaat et al., 2014: Halvorsen et al., 2019) showing some potential variations in autistic individuals. As in the previous studies (Brinkley et al., 2007; Kaat et al., 2014; Halvorsen et al., 2019), the current findings indicate that fit indices slightly improved when treating the three items describing self-injurious behaviour as a separate factor. This may indicate that the relationship between the other behaviours measured on the irritability subscale, typically aggressive and disruptive behaviours, and self-injurious behaviour, may differ for autistic individuals with intellectual disabilities compared to other populations with intellectual disabilities. However, compared to previous studies investigating the five-factor solution in samples involving autistic individuals (Brinkley et al., 2007; Halvorsen et al., 2019; Kaat et al., 2014), the current study showed similar or slightly improved fit. Since four out of five subscales demonstrated measurement invariance and the ABC is a well-established and well-researched assessment tool, it is doubtful that the improved fit of a six-factor solution is sufficient to warrant a change in the use and interpretation of the ABC. Overall, the current study justifies continued clinical use and interpretation of the original five subscales in autistic adults with intellectual disabilities.

A high number of studies indicate that the ABC is applicable in assessment and treatment evaluation for people with intellectual disabilities (Aman, 2012), including autistic individuals (Brinkley et al., 2007; Kaat et al., 2014; Halvorsen et al., 2024). Based on these and previous findings, it may be tempting to alter the factor structure according to findings in different subgroups to achieve an improved statistical fit. However, individuals with intellectual disabilities are a heterogeneous population, who often show complex and compound conditions and could be stratified into a myriad of different subgroups based on genetic findings, level of intellectual disability, presence of any co-occurring neurodevelopmental disorder, adaptive skills, communication skills, as well as any other co-occurring difficulties. All of these aspects are likely to affect the prevalence and manifestations of the behaviours measured by the ABC (Bowring et al., 2017, 2019; de Winter et al., 2011; Leader et al., 2021; McClintock et al., 2003; Melville et al., 2016; Painter et al., 2018). Developing separate scoring rules for different subgroups would be a monumental task, and it is unclear how this would be helpful in clinical settings. Moreover, many individuals with intellectual disabilities have rare disorders or rare combinations of difficulties, which increases the challenges in adapting the ABC scoring rules for specific subgroups. Altering the factor structure to the findings in the current sample resulted in only slight improvements in fit indices, an improvement that may even be specific to this specific sample.

Kaat et al. (2014) found crossover between the irritability and hyperactivity/non-compliance subscale and concluded that this was likely to be idiosyncratic to the specific sample. While cross-over was found for the same items in the current sample, the current study found additional items from the hyperactivity/non-compliance subscale that appeared to load primarily on the irritability subscale at T1, highlighting the difficulties in using single-study findings to develop alternative fit models. Moreover, community samples may differ from clinical samples with regard to the prevalence and intensity of challenging behaviours. Kaat et al. (2014) highlight that self-injurious behaviours are highly clinically salient when present. Studies focusing on autistic individuals finding these behaviours to emerge as a potential separate factor (Halvorsen et al., 2019; Kaat et al., 2014) may thus be a consequence of these behaviours being more prevalent (Steenfeldt-Kristensen et al., 2020) and thus more salient in autistic individuals, compared to non-autistic individuals with intellectual disabilities. In line with this argument, the results of the post-hoc EFAs indicate that the some aspects of the model form differed in the same sample across the three time points. This may be due to the general reduction of scores in the sample, as shown in Table 1, indicating that model form may differ with the overall level of challenging behaviours in a given sample, suggesting that model form may differ between clinical and community samples. Based on the current results, particularly the findings regarding longitudinal measurement invariance, developing more precise knowledge concerning the strengths and weaknesses of the ABC in different subgroups appear to be a more helpful approach than developing separate factor structures for different subgroups in the intellectual disability population.

The original hyperactivity/noncompliance subscale did not demonstrate longitudinal measurement invariance. These findings indicate that this subscale, comprising items describing behaviours that involve a high level of activity, restlessness, and noncompliance, are interpreted and scored differently in the same sample over time. This may be linked to the services that were provided to the patients during the study. The patient trajectories involved comprehensive assessment of mental health disorder and treatment of any diagnosed disorder, which, in over 95% of the cases, included supervision, and/or training of the patients’ primary professional caregivers (Hellerud et al., 2024; Helverschou et al., 2021). These caregivers were also often responsible for completing the ABC. It is possible that longitudinal noninvariance was caused by the informants who completed the ABC gaining new insights and understanding into the patient’s behaviours, thereby affecting their view of the patient’s behaviour and, in turn, their scoring of the ABC. Based on the post hoc EFAs, another possible explanation may be that the items on this subscale may load on different factors according to the total symptom load of the sample. In other words, the items on this subscale may be interpreted differently across time, in context of the other behaviours displayed by the individual. It is possible that the salience of these specific behaviours to observers may change according to the overall level of challenging behaviours. For instance, the individual being easily distracted is likely to be less salient to a caregiver if an individual is displaying severe aggressive or self-injurious behaviours. However, it remains a testament to the psychometric properties of the ABC that this only appeared to affect the longitudinal measurement invariance of the hyperactivity/noncompliance subscale and not the other original subscales.

The associations found for autism characteristics and communication skills are in line with previous findings of correlates of challenging behaviours (Bowring et al., 2017, 2019; Halvorsen et al., 2019), further supporting the convergent validity of the ABC subscales. When multivariate analyses for each subscale were conducted, only autism characteristics and communication skills remained significant predictors of ABC subscale scores. However, the explained variance was low, highlighting the complexity and multidimensionality of these behaviours (Bowring et al., 2017, 2019; Kildahl et al., 2023). The level of intellectual disability was reported as a dichotomous variable, which may have resulted in an underestimation of the effect of level of intellectual disability on ABC subscale scores. However, these results confirm that autism characteristics and communication skills are significantly associated with challenging behaviours in autistic individuals with intellectual disabilities, highlighting the importance of assessing these areas of functioning in the assessment of challenging behaviours and trying to disentangle their contribution to the development of challenging behaviours in each individual.

### Clinical Implications

Overall, these results indicate that continued use of the original five-factor structure of the ABC is warranted. However, these results also suggest that caution is advised in the interpretation of changes in the hyperactivity/noncompliance subscale scores over time. Thus, when using the ABC to evaluate treatment and intervention, we recommend emphasising the four subscales that showed longitudinal measurement invariance (irritability, social withdrawal, stereotypic behaviour, inappropriate speech). In addition, these findings, in line with previous findings (Halvorsen et al., 2019; Kaat et al., 2014) indicate that self-injurious behaviours may not be as closely associated with aggressive/irritable/disruptive behaviours in autistic individuals as in non-autistic individuals with intellectual disabilities. Clinicians would therefore be advised to consider these items also separately, outside of the original irritability subscale.

Measures of challenging behaviours have been found to be associated with a host of trait, state and environmental risk factors that may interact, highlighting the complexity of these behaviours. ABC scores appear to be reduced when specific mental health disorders are treated in autistic people with intellectual disabilities (Bakken et al., 2024; Hellerud et al., 2024; Helverschou et al., 2021), highlighting the need for assessment and intervention based on a comprehensive understanding of the nature, context and development of these behaviours in each individual. The ABC is a descriptive measure of the configuration and severity of these behaviours, and further measures are needed to achieve the necessary level of understanding for adapted intervention targeting the causes and triggers for these behaviours in each specific individual (Bowring et al., 2019; Kildahl et al., 2024).

### Future Research

The ABC is frequently used in studies involving the evaluation of treatment and intervention in people with intellectual disabilities, including psychopharmacological treatment (Aman, 2012; Stoddard et al., 2020). The current results indicate that such use is warranted, also for autistic people with intellectual disabilities. However, caution is advised for one of the subscales (hyperactivity/noncompliance) when using the ABC to evaluate treatment and intervention. In addition, there is a need for further research on the measurement invariance of the ABC across groups, including across autistic and non-autistic people with intellectual disabilities, as well as individuals with and without co-occurring mental health disorders.

### Limitations

The sample size of the current study was small and obtained in the specific context of units in the Norwegian healthcare system that provide specialised assessment and treatment for mental health disorder in autistic people with intellectual disabilities, limiting the generalisability of the study. That the eight centres chose potential participants may further limit the generalisability of the sample. However, the sample also included individuals with severe and complex symptom presentations, i.e. individuals who may frequently be excluded from research (Helverschou et al., 2021). Moreover, because the current sample was obtained from autistic individuals with co-occurring intellectual disabilities referred for mental health assessment, these findings are not generalisable to autistic people with intellectual disabilities in general.

Due to the lack of a comparison group, it is not possible to draw inferences concerning comparative fit of the model based on the current data. Moreover, the statistical fit of the original five-factor model was not optimal, and a six-factor solution only showed marginally improved fit. Despite lack of optimal fit, however, 4/5 subscales showed longitudinal measurement invariance, indicating that these are suited for within-subject comparisons despite the non-optimal fit.

As professional caregivers completed the ABC for most of the participants, it is possible that different caregivers completed the ABC for the same participant at the three time points, which may have affected the scores. Finally, there was some attrition in the sample from T1, which may have affected model fit and other results. However, the participants who were only included at T1 only differed from the remaining sample with regard to gender, and not with regard to ABC subscale scores or scores on other variables.

## Conclusions

The current study confirms the applicability of the ABC in autistic individuals with intellectual disabilities for the assessment of challenging behaviours, as well as for the evaluation of treatment and intervention. However, these findings also highlight that caution is advised for one of its subscales when making comparisons in the same individuals across time (hyperactivity/noncompliance).

## Appendix

**Appendix 1:** Post-hoc Exploratory Factor Analysis, T1 Data (N = 200), ABC Item Loadings

Item number, short form, original factor (F1-F5)	Component/Factor loading						
	F1	F2	F3	F4	F5	F6	F7
18. Disobedient (F4)	.80						
57. Temper tantrums (F1)	.78						
14. Irritable (F1)	.73						
4. Aggressive (F1)	.72						
10. Tantrums (F1)	.71						
21. Disturbs others (F4)	.70						
7. Boisterous (F4)	.69						
24. Uncooperative (F4)	.68						
28. Not pay attention (F4)	.67						
36. Mood changes (F1)	.66					.34	
8. Screams (F1)	.63						
47. Stamps feet (F1)	.63					.36	
31. Disrupts group (F4)	.61						
41. Cries and Screams (F1)	.61						
51. Pays no attention (F4)	.60				.41		
29. Demands (F1)	.60						
56. Ignores directions (F4)	.59						
13. Impulsive (F4)	.57						
19. Yells (F1)	.56						
34. Cries (F1)	.39						
39. Not sit still (F4)		.88					
54. Excessively active (F4)	.31	.82					
15. Restless (F4)		.78					
38. Not stay in seat (F4)		.76					
1. Active (F4)	.41	.73					
48. Runs or jumps (F4)	.45	.66					
5. Seeks isolation (F2)			.93				
30. Isolates self (F2)			.93				
42. Prefers alone (F2)			.86				
16. Withdrawn (F2)			.79				
25. Depressed mood (F1)			.50				
26. Resists contact (F2)			.42	.31			
58. Few social reactions (F2)			.40		.35		
55. Responds neg. to affect. (F2)			.36	.34			
35. Repetitive hand movem. (F3)				.85			
11. Repetitive movements (F3)				.78			
27. Body movements (F3)				.63			
45. Waves extremities (F3)				.66			
6. Body movements (F3)				.69			
49. Rocks body (F3)				.49			
17. Odd behavior (F3)	.44			.47			
53. Inactive (F2)		−.33			.65		
37. Unresponsive (F2)					.60		
32. Stays in one position (F2)					.56		
40. Difficult to get through (F2)					.55		
43. Does not communicate (F2)					.51		
3. Sluggish (F2)			.35		.50		
20. Lacks emotional response (F2)			.32	.30	.43		
12. Preoccupied (F2)					.41		
23. Watches others (F2)					.40		
2. Injures self (F1)	.36					.84	
50. Hurts self (F1)	.45					.83	
52. Self-violence (F1)	.41					.81	
46. Repeats words (F5)							.86
22. Repetitive speech (F5)							.80
9. Talks excessively (F5)	.40						.62
33. Talks to self (F5)							.54

Note The table shows factor loadings (regression coefficients) between the items and the standardized factors. Only coefficients >.30 are included in the table, retrieved from the pattern matrix. One item did not load >.30 on any of the seven factors: 44. Easily distracted (F4)

**Appendix 2:** Post-hoc Exploratory Factor Analysis, T2 Factor Loadings (N = 169)

Item number, short form, original factor (F1-F5	Component/Factor loading						
	F1	F2	F3	F4	F5	F6	F7
57. Temper tantrums (F1)	.78				.32		
10. Tantrums (F1)	.75				.35		
14. Irritable (F1)	.74						
18. Disobedient (F4)	.70	.44					
36. Mood changes (F1)	.64				.36		
4. Aggressive (F1)	.63						
7. Boisterous (F4)	.63	.37					.33
41. Cries and Screams (F1)	.60						
56. Ignores directions (F4)	.60						
51. Pays no attention (F4)	.59	.47					
21. Disturbs others (F4)	.58	.35					
8. Screams (F1)	.58						
47. Stamps feet (F1)	.58				.47		
31. Disrupts group (F4)	.57	.44		.30			
24. Uncooperative (F4)	.54	.40	.32				
13. Impulsive (F4)	.52	.48					
29. Demands (F1)	.51						
28. Not pay attention (F4)	.50	.41					
19. Yells (F1)	.48				.35		
34. Cries (F1)	.44	−.31		.32			
39. Not sit still (F4)		.80					
15. Restless (F4)		.76					
1. Active (F4)		.75					
38. Not stay in seat (F4)		.73					
54. Excessively active (F4)		.71					
40. Difficult to get through (F2)		.54				.35	
48. Runs or jumps (F4)	.44	.50		.47			
43. Does not communicate (F2)		.49				.36	
12. Preoccupied (F2)		.39				.45	
5. Seeks isolation (F2)			.94				
30. Isolates self (F2)			.94				
16. Withdrawn (F2)			.86				
42. Prefers alone (F2)			.82				
26. Resists contact (F2)			.50				
25. Depressed mood (F1)			.45				
58. Few social reactions (F2)			.42				
20. Lacks emotional response (F2)		.38	.39			.38	
55. Responds neg. to affect. (F2)			.38				
3. Sluggish (F2)			.35				
35. Repetitive hand movem. (F3)				.85			
6. Body movements (F3)				.78			
11. Repetitive movements (F3)				.76			
45. Waves extremities (F3)				.73			
27. Body movements (F3)				.59		.36	
49. Rocks body (F3)				.58			
17. Odd behavior (F3)		.34		.36			.33
50. Hurts self (F1)	.30				.87		
52. Self-violence (F1)	.34				.85		
2. Injures self (F1)					.79		
23. Watches others (F2)						.70	
53. Inactive (F2)						.72	
32. Stays in one position (F2)						.63	
37. Unresponsive (F2)						.50	
12. Preoccupied (F2)		.39				.45	
9. Talks excessively (F5)							.88
22. Repetitive speech (F5)							.63
46. Repeats words (F5)				.32	.31		.56
33. Talks to self (F5)	.35		.32				.45

Note The table shows factor loadings (regression coefficients) between the items and the standardized factors. Only coefficients >.30 are included in the table, retrieved from the pattern matrix

**Appendix 3:** Post-hoc exploratory factor analysis, T3 data (N = 169), item loadings

Item number, short form, original factor (F1-F5)	Component/Factor loading				
	F1	F2	F3	F4	F5
54. Excessively active (F4)	.90				
1. Active (F4)	.85				
15. Restless (F4)	.69				
39. Not sit still (F4)	.67				
21. Disturbs others (F4)	.65			.34	
7. Boisterous (F4)	.64				
13. Impulsive (F4)	.59				
31. Disrupts group (F4)	.54				
19. Yells (F1)	.54			.35	
48. Runs or jumps (F4)	.53				.40
38. Not stay in seat (F4)	.51	.41			
29. Demands (F1)	.50	.37	.33		
9. Talks excessively (F5)	.48				
22. Repetitive speech (F5)	.42		.33		
44. Easily distracted (F4)	.32				
53. Inactive (F2)	−.36				
18. Disobedient (F4)	.46	.64			
40. Difficult to get through (F2)		.63	.31		.31
37. Unresponsive (F2)		.62	.32		
41. Cries and Screams (F1)	.34	.58			
43. Does not communicate (F2)		.57			
14. Irritable (F1)		.56			
56. Ignores directions (F4)		.54			
32. Stays in one position (F2)		.54	.40		
10. Tantrums (F1)		.53		.50	
28. Not pay attention (F4)	.35	.52	.40		
36. Mood changes (F1)	.42	.50		.47	
51. Pays no attention (F4)	.34	.49	.34		
57. Temper tantrums (F1)	.48	.49		.36	
8. Screams (F1)	.37	.47			.34
23. Watches others (F2)		.44			
33. Talks to self (F5)		.38			
30. Isolates self (F2)			.99		
5. Seeks isolation (F2)			.79		
42. Prefers alone (F2)			.79		
16. Withdrawn (F2)			.76		
3. Sluggish (F2)	−.34	.31	.58		
55. Responds neg. to affect. (F2)		.37	.49		
25. Depressed mood (F1)		.33	.46		
24. Uncooperative (F4)	.41	.39	.43		
26. Resists contact (F2)			.40		
2. Injures self (F1)				.91	
52. Self-violence (F1)				.91	
50. Hurts self (F1)				.89	
47. Stamps feet (F1)	.38	.39		.45	
4. Aggressive (F1)	.40	.34		.41	
27. Body movements (F3)				.36	.32
11. Repetitive movements (F3)					.76
6. Body movements (F3)					.74
35. Repetitive hand movem. (F3)				.37	.67
45. Waves extremities (F3)					.63
17. Odd behavior (F3)			.35	.32	.48
58. Few social reactions (F2)		.43	.40		.43
49. Rocks body (F3)	.30		.30		.43
12. Preoccupied (F2)		.34			.43
20. Lacks emotional response (F2)		.38			.39
46. Repeats words (F5)	.33				.38

Note The table shows factor loadings (regression coefficients) between the items and the standardized factors. Only coefficients >.30 are included in the table, retrieved from the pattern matrix. One item did not show loadings >.30 on any factor: 34. Cries (F1)
